# Actinomycosis presenting as an isolated pleural effusion in a patient with an HIV infection: a case report and literature review

**DOI:** 10.1186/s12981-021-00412-5

**Published:** 2021-11-17

**Authors:** Jung Wan Park, Yon Hee Kim, Eunjung Lee, Se Yoon Park, Tae Hyong Kim

**Affiliations:** 1 Department of Internal Medicine, Soonchunhyang University Seoul Hospital, Soonchunhyang University College of Medicine, Seoul, Republic of Korea; 2 Department of Internal Medicine, Soonchunhyang University Cheonan Hospital, Soonchunhyang University College of Medicine, Cheonan, Republic of Korea; 3 Department of Pathology, Soonchunhyang University Seoul Hospital, Soonchunhyang University College of Medicine, Seoul, Republic of Korea

**Keywords:** Human immunodeficiency virus, Actinomycosis, Pleural effusion

## Abstract

**Background:**

Thoracic actinomycosis is an uncommon, chronic, and progressive infection, especially in patients with HIV. We report a case of thoracic actinomycosis presenting as an isolated pleural effusion in a patient with an HIV infection.

**Case presentation:**

A 68-year-old patient with progressive dyspnea and fever was admitted. On the right side, an ipsilateral massive pleural effusion was confirmed on the chest radiograph, and an HIV infection was newly diagnosed. A pleural biopsy was performed for the further differential diagnosis of potential opportunistic infections and malignancies. The pathology findings were consistent with actinomycosis.

**Conclusions:**

Active diagnostic approaches such as a pleural biopsy should be considered for indeterminate pleural effusions in immunocompromised patients.

## Background

Actinomycosis is a relatively rare, chronic, and progressive infection caused by *Actinomyces* species, which are part of the human mucosal flora. Thoracic involvement with *Actinomyces* constitutes approximately 15% of the patients diagnosed with actinomycosis and an isolated pleural effusion is a rare clinical presentation of thoracic actinomycosis [1, 2]. We report a rare case of thoracic actinomycosis presenting as an isolated pleural effusion in a newly diagnosed HIV-infected patient along with a literature review.

## Case presentation

A 68-year-old man with a history of smoking and without comorbid conditions was admitted to the emergency department on February 28, 2021, with a 15-day history of progressive and worsening dyspnea. He also reported chills and a productive cough. His medical history included herpes zoster and an inguinal hernia. He had no history of thoracic traumatic injury or any other surgeries.

The patient’s body temperature at the time of admission was 39.0 °C, with a heart rate of 141 beats/min and blood pressure of 174/107 mmHg. The patient’s oxygen saturation was 77% while breathing ambient air. The physical examination was remarkable for decreased lung sounds and wheezy breathing sounds with rales over the entire right lung. Laboratory tests revealed a white blood cell count of 20.000/mm^3^, a hemoglobin level of 11.8 g/dL, a platelet count of 455 × 10^3^/μL, a C-reactive protein level of 34.6 mg/dL, and a procalcitonin level of 59.42 ng/mL. The HIV antigen/antibody combination test revealed a positive result and the real-time polymerase chain reaction (PCR) test for HIV was 1.11 × 10^5^ copies/mL. The CD4 + T-lymphocyte cell count was 308 cells/µL (15%).

**Fig. 1:**
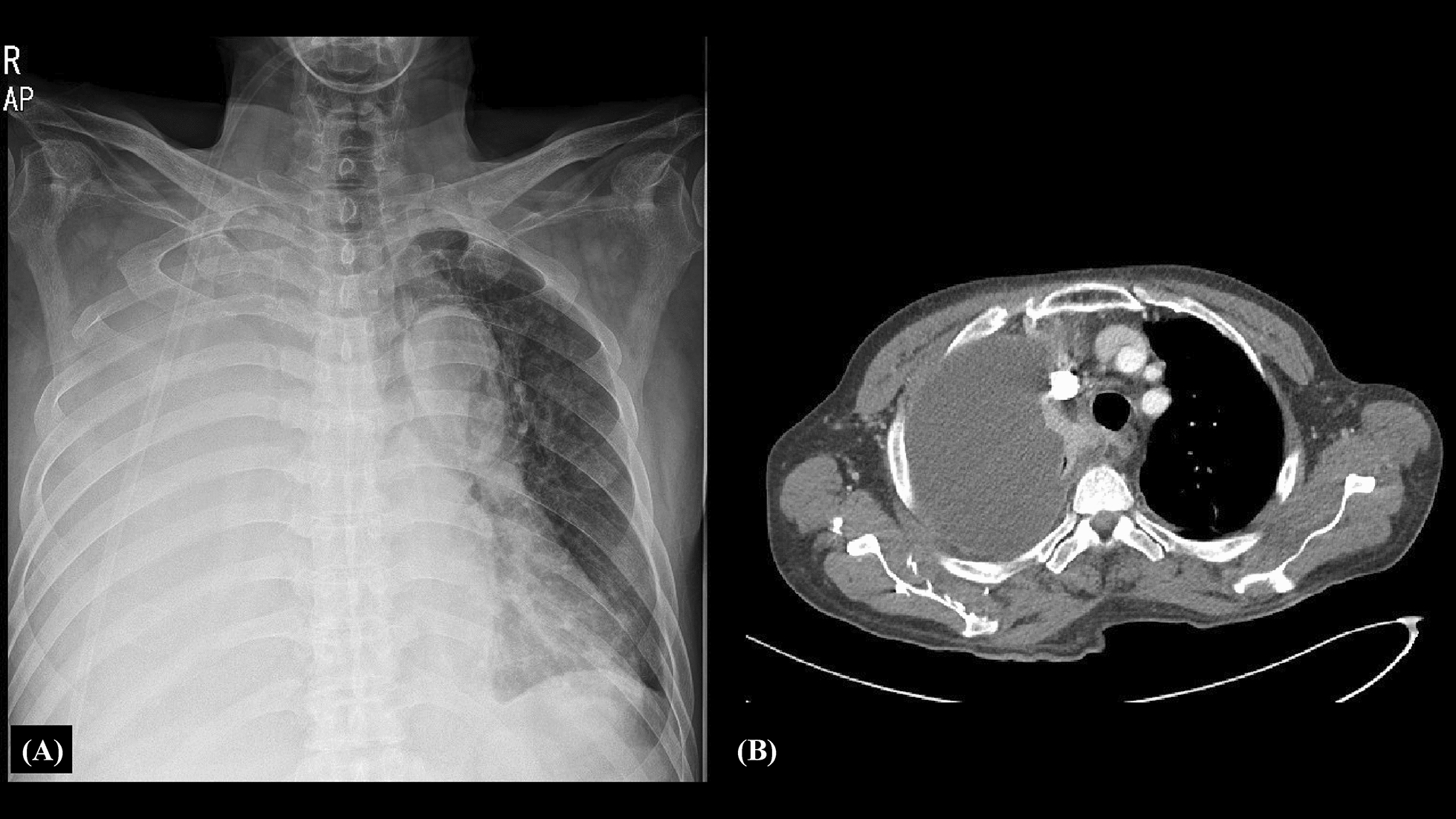
**A**, **B** Chest radiograph and chest computed tomography. The chest radiography showed a totally whited-out right lung and chest computed tomography revealed a large amount of right pleural effusion and diffuse pleural enhancement

**Fig. 2:**
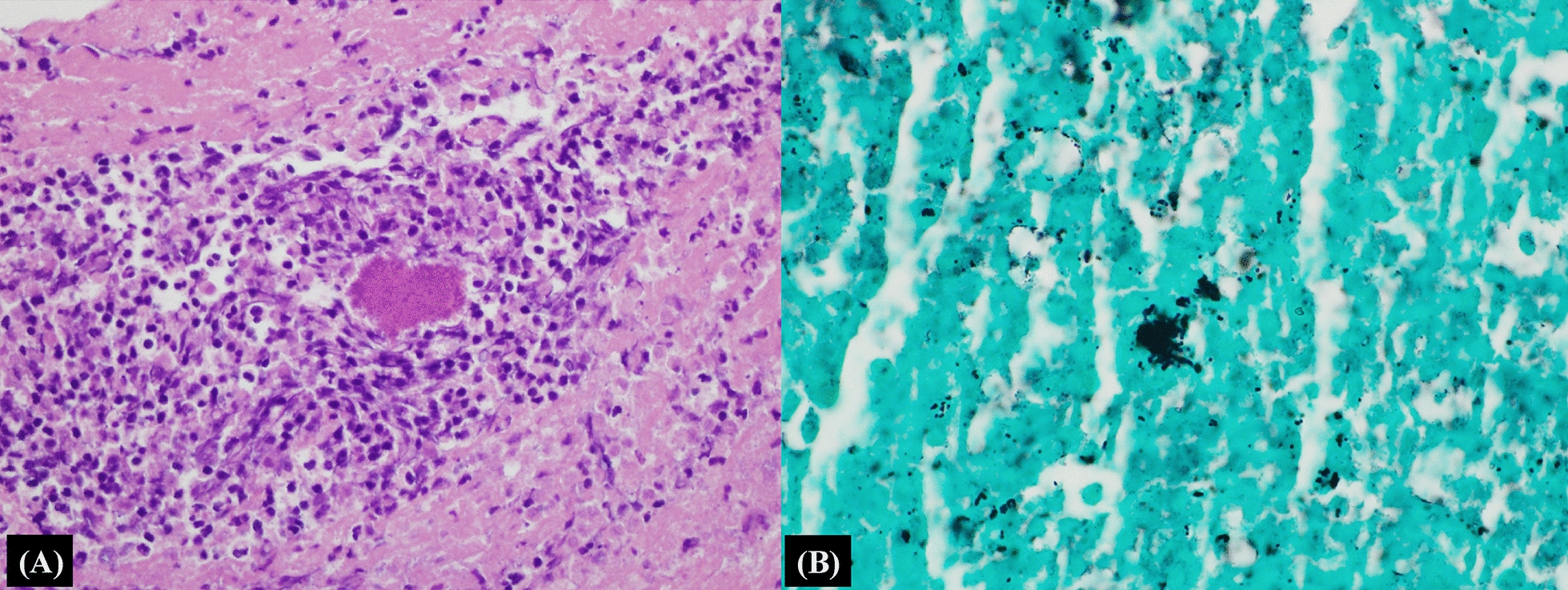
**A** Histologic examination: hematoxylin and eosin (H & E) staining (× 400). A bacterial colony (sulfur granule) is found at the center of mixed inflammatory cells. **B** Grocott methenamine silver stain (GMS) stain (× 1000). The GMS stain demonstrates filamentous bacteria

Chest radiography showed a totally whited-out right lung and chest computed tomography (CT) revealed a large amount of right pleural effusion, diffuse pleural enhancement, and multifocal low dense lesions in the right lobe (Fig. 1A: chest radiograph, B: chest CT). Percutaneous catheter drainage of pleural fluid and pleural fluid analysis were done on the third day after admission to relieve dyspnea and make a differential diagnosis. Pleural fluid analysis revealed an exudate of white blood cells (3860/μL) and an adenosine deaminase (ADA) level of 179 IU/L. The white blood cell differential count was not available due to multiple lysed cells. Cytologic examination of the pleural effusion and sputum revealed several acute inflammatory cells and necrotic cellular debris without microorganisms. No malignant cells were found in the pleural fluid. Based on the high level of pleural ADA and the newly diagnosed HIV infection, a presumptive diagnosis of tuberculosis (TB) pleurisy was made. Anti-tuberculosis treatment was initiated, although the acid-fast bacilli (AFB) stain and culture results, as well as tuberculosis real-time PCR analysis of the pleural fluid, were negative. The TB-specific antigen interferon (INF)-gamma was negative. A pleural biopsy was performed on day 6 after admission, which revealed two tiny fragments of pleura showing abundant acute and chronic inflammatory cell infiltrates with ovoid basophilic radiating filaments under light microscopy, consistent with actinomycosis (Fig. 2). Anti-tuberculosis medications were stopped. Intravenous ampicillin (200 mg/kg/day) was administered and anti-retroviral therapy (tenofovir alafenamide fumarate/emtricitabine/bictegravir) was initiated. After 4 weeks of parenteral ampicillin, the patient was switched to oral amoxicillin for 11 months. Chest radiography performed during a follow-up 15 months later revealed a small amount of loculation in the right hemithorax. The patient came to the hospital on September 28, 2021, and did not complain of any special symptoms. There were no abnormal findings in most of the examination results including the chest X-ray.

## Discussion and conclusion

A unique case of isolated pleural actinomycosis was confirmed in an HIV patient without a history of thoracic trauma or surgery. The clinical presentation of the patient in this case was fever, progressive dyspnea, and unilateral pleural thickening with massive effusion. In this case, only an aggressive pleural biopsy provided a timely diagnosis and guided the actinomycosis treatment.

The main symptoms of thoracic actinomycosis were cough expectoration, blood-stained sputum, hemoptysis, fever, and chest pain [1, 3]. Radiographically, thoracic actinomycosis may involve either lung and show multiple cavitary lesions. A mass lesion or pneumonia with or without pleural involvement is usual and pleural thickening, effusion, or empyema is found in more than 50% of the cases [4]. Otherwise, an isolated pleural effusion is an extremely rare clinical presentation of thoracic actinomycosis. In addition, an organism is not often isolated from a pleural effusion. The previously reported cases showed the diverse clinical features of an isolated pleural effusion in thoracic actinomycosis, but the cases were generally associated with structural injury or thoracic surgery [5–8]. However, the patient in our case had no history of thoracic trauma or surgery and had a newly diagnosed HIV infection.

Only four case reports in patients with HIV have been published [9–12]. We reviewed a total of five case reports involving thoracic actinomycosis in patients with an HIV infection, including our patient (Table 1). It was difficult to determine the patients’ immunity levels because of inappropriate CD4 count reporting. However, three patients including our patient, received anti-retroviral therapy (ART). Most patients were first suspected to have other diseases (e.g., pulmonary tuberculosis, lung mass, *Pneumocystis jiroveci* pneumonia) and were treated accordingly. Nevertheless, symptom improvement was slow, and thoracic actinomycosis was confirmed via sulfur granules or *Actinomyces* species detected in trans-bronchial lung biopsy (TBLB) or bronchoalveolar lavage (BAL) samples. All patients were treated with intravenous penicillin G or peroral amoxicillin, and the duration of treatment varied from 3 weeks to 12 months. The patient in our case was also successfully treated for 12 months. All but one patient improved. The single patient death was attributed not to thoracic actinomycosis but superimposed cryptococcal meningitis.

**Table 1:** Studies reporting cases of thoracic actinomycosis associated with HIV infection

Case no	Year	Age/sex	Symptom	CD4 count	Combined disease	ART	Radiologic finding	Confirmatory specimen	Treatment	Treatment duration	Outcome
1	2017	52/M	Massive hemoptysis	Data not available	HCV	Zidovudine, lamivudine 150 mg BD and efavirenz 600 mg daily	HRCT: Large mass-like consolidation in left upper lobe with central necrosis and excavation and also adjacent nodular infiltration	TBLB	Lobectomy + PO amoxicillin + clindamycin, metronidazole, trimethoprim/sulfamethoxazole (TMP-SMX) and ceftriaxone	6 months	Improved
2	1997	41/M	Asymptomatic	340/mm^3^	HCV, alcohol dependency	Zidovudine (100 mg qid) + ranitidine (150 mg at night)	HRCT: numerous nodular densities < 5 mm in diameter on the right side more than on the left	BAL	IV penicillin G	21 days	Improved
									PO Ampicillin	6 months	
3	1989	42/M	Fever, productive cough, pleuritic chest pain	Data not available	IV drug abuser	Data not available	Chest x-ray: bilateral patchy alveolar infiltration	TBLB	IV penicillin G	3 weeks	Improved
4	1993	47/M	Fever, cough	Data not available	–	Data not available	Chest x-ray: lingular infiltration	BAL	IV penicillin G	4 weeks	Expired
5		68/M	Dyspnea, productive cough, chills	308/mm^3^	–	Tenofovir alafenamide Fumarate/emtricitabine/bictegravir	Chest CT: large amount of right pleural effusion, diffuse pleural enhancement	Pleural biopsy	IV ampicillin	4 weeks	Improved
									PO amoxicillin	11 months	

Most patients showed symptoms and radiological findings indistinguishable from pneumonia or tuberculosis and were confirmed by biopsy. The patients were treated for various durations ranging from 3 weeks to 12 months, and all but one patient improved

Cases of actinomycosis have been reported in the setting of immunocompromised conditions including HIV infections [13]. Actinomycosis combined with immunosuppression is associated with higher mortality than immunosuppression alone. A multicenter retrospective study conducted in Paris from 1997 to 2009 showed death in 21% of all cases of invasive actinomycosis and was associated with immunocompromised status (p = 0.027)[14]. Thus, the recognition of actinomycosis is a more important issue in immunocompromised patients such as those with HIV infections. However, actinomycosis is uncommon, with limited clinical experience, and laboratory investigations are challenging [15]. Therefore, a broad differential diagnosis with uncommon manifestations should be considered and active diagnostic approaches are needed.

In conclusion, isolated pleural effusion is a rare clinical presentation of thoracic actinomycosis. However, actinomycosis can be fatal in immunocompromised patients such as HIV-infected individuals. Active diagnostic approaches such as a pleural biopsy should be considered in immunocompromised patients with an indeterminate pleural effusion.

## Data Availability

The data that support the findings of this study are openly available.

## Abbreviations

HIV: Human immunodeficiency virus
TB: Tuberculosis
ART: Antiretroviral therapy
TBLB: Transbronchial lung biopsy
BAL: Bronchoalveolar lavage
ADA: Adenosine deaminase
PCR: Polymerase chain reaction
CT: Computed tomography
INF: Interferon
